# Supplementary material for: Correlation between Blunted Nocturnal Decrease in Diastolic Blood Pressure and Oxidative Stress: An Observational Study
Source: Antioxidants (Basel). 2022 Dec 9;11(12):2430. doi: 10.3390/antiox11122430 (PMC9774450; doi:10.3390/antiox11122430)
Supplement: Supplementary file 1 [file antioxidants-11-02430-s001.zip › antioxidants-2061505-supplementary.pdf]

Table S1. Binary logistic regression models for the presence of a non-dipper DBP profile based on each of the evaluated redox parameters.

Variables	B	*P*-value	Exp(B)	95%CI	
				Inferior	Superior
**TBARS**. *Model summary: P-value (F-test)< 0.001, P-value (Hosmer and Lemeshow)= 0.523, -2LL= 202.2, R^2 (Nagelkerke)= 0.293, Overall accuracy= 0.79.*					
nSBP (mmHg)	0.037	0.075	1.038	0.996	1.081
nDBP (mmHg)	0.056	0.059	1.057	0.998	1.120
Diuretic use (yes)	1.004	0.007	2.728	1.308	5.692
TBARS (nmol/mg)	0.738	0.002	2.091	1.319	3.316
**Reduced thiols.** *Model summary: P-value (F-test)< 0.001, P-value (Hosmer and Lemeshow)= 0.095, -2LL= 204.9, R^2 (Nagelkerke)= 0.274, Overall accuracy= 0.79.*					
Alcohol intake (yes)	0.732	0.082	2.080	0.912	4.744
nSBP (mmHg)	0.039	0.060	1.040	0.998	1.083
nDBP (mmHg)	0.040	0.099	1.050	0.991	1.113
Diuretic use (yes)	0.636	0.085	1.889	0.916	3.895
Reduced thiols (µmol/mg)	-1.772	0.042	0.170	0.031	0.939
**Vitamin E.** *Model summary: P-value (F-test)< 0.001, P-value (Hosmer and Lemeshow)= 0.331, -2LL= 202.8, R^2 (Nagelkerke)= 0.269, Overall accuracy= 0.78.*					
Alcohol intake	0.863	0.044	2.370	1.022	5.498
nSBP (mmHg)	0.039	0.061	1.039	0.998	1.082
nDBP (mmHg)	0.050	0.091	1.051	0.992	1.114
Diuretic use	0.653	0.077	1.922	0.931	3.967
Vitamin E (µg/mg)	-0.042	0.094	0.959	0.913	1.007
**Vitamin A.** *Model summary: P-value (F-test)< 0.001, P-value (Hosmer and Lemeshow)= 0.280, -2LL= 199.2, R^2 (Nagelkerke)= 0.289, Overall accuracy= 0.78.*					
Alcohol intake (yes)	0.997	0.024	2.710	1.142	6.435
nSBP (mmHg)	0.038	0.066	1.039	0.998	1.082
nDBP (mmHg)	0.052	0.078	1.054	0.994	1.117
Diuretic use (yes)	0.740	0.047	2.095	1.009	4.351

Vitamin A (µg/mg)	-1.225	0.016	0.294	0.109	0.792
**Copper.** *Model summary: P-value (F-test)< 0.001, P-value (Hosmer and Lemeshow)= 0.513, -2LL= 210.6, R^2 (Nagelkerke)= 0.243, Overall accuracy= 0.78.*					
Age (years)	0.045	0.014	1.046	1.009	1.084
nDBP (mmHg)	0.112	< 0.001	1.118	1.073	1.165
Copper (µg/dL)	0.017	0.049	1.017	1.001	1.035
**TBARS/Thiol ratio.** *Model summary: P-value (F-test)< 0.001, P-value (Hosmer and Lemeshow)= 0.625, -2LL= 200.5, R^2 (Nagelkerke)= 0.296, Overall accuracy= 0.80.*					
Diuretic use (yes)	0.823	0.025	2.276	1.106	4.684
nDBP (mmHg)	0.096	< 0.001	1.101	1.059	1.144
TBARS/Thiol ratio	0.475	< 0.001	1.609	1.246	2.075
**TBARS/Vitamin E ratio.** *Model summary: P-value (F-test)< 0.001, P-value (Hosmer and Lemeshow)= 0.377, -2LL= 190.9, R^2 (Nagelkerke)= 0.312, Overall accuracy= 0.78.*					
Alcohol intake (yes)	0.821	0.061	2.273	0.964	5.357
Diuretic use (yes)	0.677	0.076	1.967	0.931	4.159
nSBP (mmHg)	0.038	0.073	1.039	0.996	1.083
nDBP (mmHg)	0.051	0.089	1.053	0.992	1.117
TBARS/Vitamin E ratio	0.135	0.002	1.144	1.051	1.246
**TBARS/Vitamin A ratio.** *Model summary: P-value (F-test)< 0.001, P-value (Hosmer and Lemeshow)= 0.135, -2LL= 188.3, R^2 (Nagelkerke)= 0.326, Overall accuracy= 0.82.*					
Alcohol intake (yes)	0.967	0.029	2.631	1.106	6.258
Diuretic use (yes)	0.740	0.056	2.096	0.981	4.481
nDBP (mmHg)	0.093	< 0.001	1.098	1.054	1.143
TBARS/Vitamin A ratio	0.896	< 0.001	2.450	1.578	3.805

DBP—Diastolic blood pressure. TBARS—Thiobarbituric acid reactive substances. nSBP—Nocturnal systolic blood pressure. nDBP—Nocturnal DBP. *-2LL*—-2*log-likelihood. R^2—Determination coefficient.
